# Sperm-borne phospholipase C zeta-1 ensures monospermic fertilization in mice

**DOI:** 10.1038/s41598-018-19497-6

**Published:** 2018-01-22

**Authors:** Kaori Nozawa, Yuhkoh Satouh, Takao Fujimoto, Asami Oji, Masahito Ikawa

**Affiliations:** 1 Research Institute for Microbial Diseases, Osaka University, Suita, Osaka 5650871 Japan; 2 Graduate School of Medicine, Osaka University, Suita, Osaka 5650871 Japan; 3 Graduate School of Pharmaceutical Sciences, Osaka University, Suita, Osaka 5650871 Japan; 4 The Institute of Medical Science, The University of Tokyo, Tokyo, 1088639 Japan

## Abstract

Sperm entry in mammalian oocytes triggers intracellular Ca^2+^ oscillations that initiate resumption of the meiotic cell cycle and subsequent activations. Here, we show that phospholipase C zeta 1 (PLCζ1) is the long-sought sperm-borne oocyte activation factor (SOAF). *Plcz1* gene knockout (KO) mouse spermatozoa fail to induce Ca^2+^ changes in intracytoplasmic sperm injection (ICSI). In contrast to ICSI, *Plcz1* KO spermatozoa induced atypical patterns of Ca^2+^ changes in normal fertilizations, and most of the fertilized oocytes ceased development at the 1–2-cell stage because of oocyte activation failure or polyspermy. We further discovered that both zona pellucida block to polyspermy (ZPBP) and plasma membrane block to polyspermy (PMBP) were delayed in oocytes fertilized with *Plcz1* KO spermatozoa. With the observation that polyspermy is rare in astacin-like metalloendopeptidase *(Astl)* KO female oocytes that lack ZPBP, we conclude that PMPB plays more critical role than ZPBP *in vivo*. Finally, we obtained healthy pups from male mice carrying human infertile *PLCZ1* mutation by single sperm ICSI supplemented with *Plcz1* mRNA injection. These results suggest that mammalian spermatozoa have a primitive oocyte activation mechanism and that PLCζ1 is a SOAF that ensures oocyte activation steps for monospermic fertilization in mammals.

## Introduction

At mammalian fertilization, sperm entry causes Ca^2+^ oscillations, repetitive acute increases and decreases in cytosolic Ca^2+^ levels lasting for several hours in human and mouse eggs, which trigger not only resumption of the meiotic cell cycle but also block polyspermy, the entry of multiple sperm heads into the ooplasm^1^. These events are collectively referred to as oocyte activation and are prerequisite for normal subsequent mitosis and embryonic development^2^.

The mechanism of how sperm generate the Ca^2+^ transients in oocytes have been studied for decades. Microinjection studies introducing extracts from rabbit, hamster, or boar spermatozoa have strongly implied that, in mammals, protein(s) in sperm soluble fraction can trigger Ca^2+^ oscillations comparable to those caused by fertilization^3,4^. Such proteins are termed as sperm-borne oocyte activation factor (SOAF). So far, multiple candidates for SOAF have been raised by microinjection studies^5,6^. However, injection studies of purified protein^7,8^ or analysis of gene knockout (KO) mice^9^ showed discrepancies in identifying the SOAF.

A sperm-specific phospholipase C zeta 1 (PLCζ1) was identified as a candidate for SOAF^10^. In somatic cells, phospholipase C (PLC) mediates the digestion of phosphatidylinositol 4,5-bisphosphate (PIP_2_) to generate inositol triphosphate (IP_3_). These cause the release of Ca^2+^ from the endoplasmic reticulum^11^. PLCζ1 was also shown that it can induce Ca^2+^ oscillations in oocytes by releasing Ca^2+^ from the oocyte’s endoplasmic reticulum at levels far above the unstimulated very low basal intracytoplasmic Ca^2+^ concentrations^12^. However, *Plcz1* KO mouse was reported preliminary at a conference as male infertile because of defective spermatogenesis^13^.

In this report, we generated *Plcz1* KO mice with the CRISPR/Cas9 system and revealed that PLCζ1 plays an essential role in ICSI fertilization but not in normal fertilization. While no Ca^2+^ spikes were observed in ICSI fertilized oocytes, atypical Ca^2+^ spikes were observed in all oocytes from normal fertilization. Very recently, Hachem *et al*., generated *Plcz1* KO mice and reported similar results, but there are significant discrepancies between that report and our data regarding Ca^2+^ spikes^14^. We further discovered that the PMBP plays a more critical role than ZPBP *in vivo*. Our findings revealed the existence of a PLCζ1 independent oocyte activation mechanism and clarified the role of PLCζ1 in ensuring monospermic fertilization.

## Results

### Generation of *Plcz1* KO mice and oocyte activation in micromanipulation

Because conventional gene knockout approaches usually replace one to several exons with a drug-resistant gene cassette, there remains a risk of unexpected effects from the truncated open reading frames and the exogenously introduced promoter^15^. To exclude such possible artifacts, here we utilized a CRISPR/Cas9 approach that deleted all the coding exons (exons 2–14) to generate *Plcz1* KO mice (Supplementary Fig. 1). Absence of the PLCζ1 protein was confirmed by western blotting analysis. Examination of our *Plcz1* KO mice did not show any defects in spermatogenesis, sperm morphology, motility, or acrosome reaction rates (Supplementary Fig. 1).

**Figure 1:**
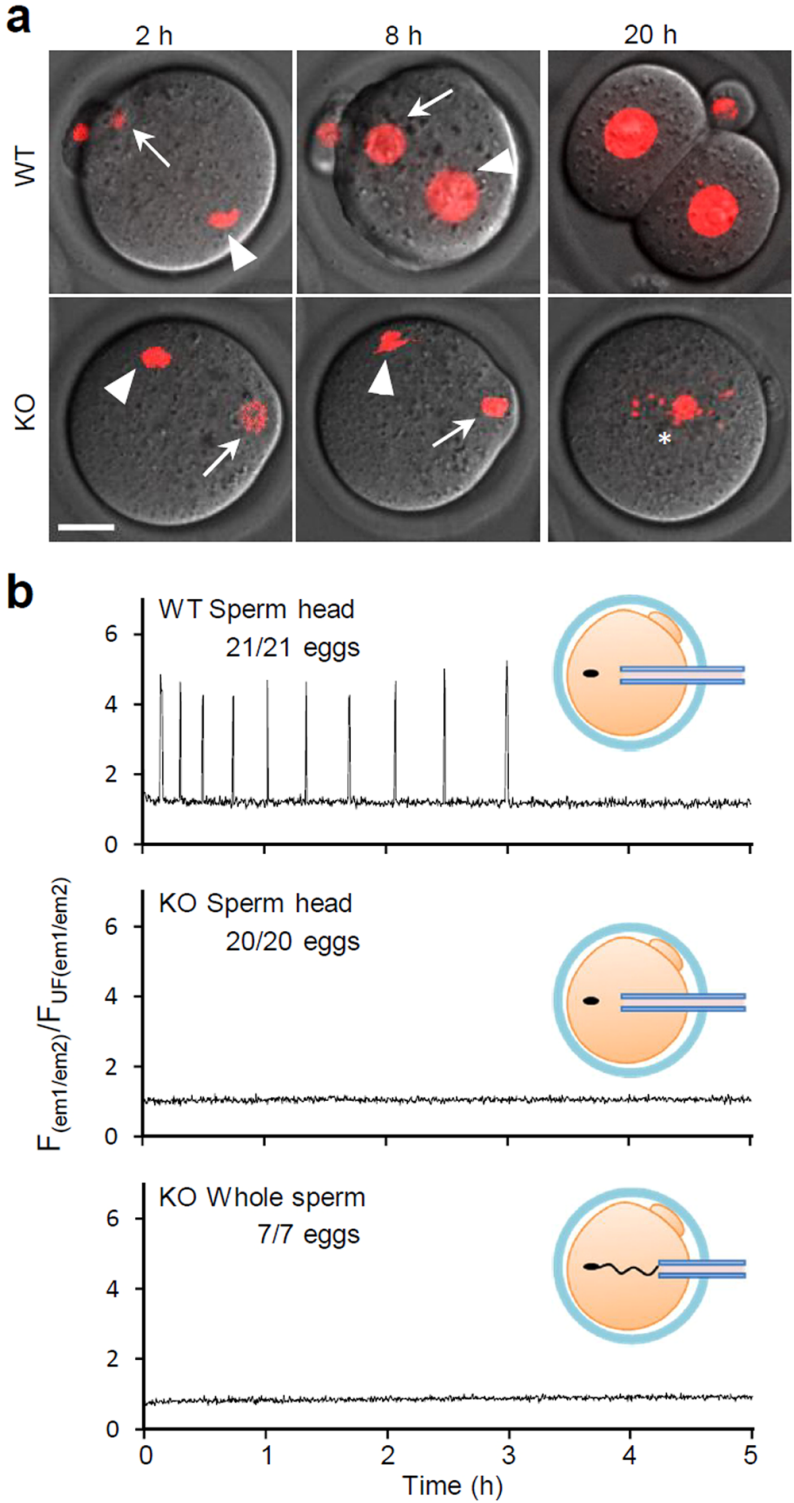
PLCζ1 is the essential SOAF in ICSI. (**a**) Oocytes at 2, 8, and 20 h after ICSI. Nuclei/pronuclei (PN) were visualized with H2B-mCherry (red). Oocytes with PN (WT 69/69 vs. KO 1/62), and 2cell embryos (WT 53/69 vs. KO 0/62) were counted at 8 h and 20 h after ICSI, respectively. Representative images are shown. Arrows, arrowheads, and asterisk indicate maternal, paternal, and fragmented chromosomes, respectively. Scale bar = 20 μm. (**b**) Intracellular Ca^2+^ changes in oocytes after ICSI. Ratio of GEM-GECO fluorescence was recorded.

To examine the oocyte activation abilities of *Plcz1* KO spermatozoa, we performed intracytoplasmic sperm injection (ICSI) using epididymal spermatozoa. All oocytes injected with wild-type (WT) sperm heads formed pronuclei (PN), and most of them developed to 2-cell embryos (100% and 76.8%, respectively, *n* = 69; Fig. 1a). In contrast, oocytes injected with *Plcz1* KO sperm heads failed to form PN or develop to 2-cell stage embryos (1.6% and 0%, respectively, *n* = 62; Fig. 1a), similarly to sham ICSI treatments (3.3% and 3.3%, respectively, *n* = 61; Supplementary Fig. 2a). Recordings of intracellular Ca^2+^ changes in these ICSI oocytes revealed that *Plcz1* KO sperm heads lost the ability to induce rises in intracellular Ca^2+^ (Fig. 1b). No spikes were observed even after up to three sperm heads were injected (Supplementary Fig. 2b). Injection of whole spermatozoa also resulted in quiescent oocytes indicating no SOAF activity remained (Fig. 1b). These data strongly support the idea that PLCζ1 is the SOAF.

### Fecundity of *Plcz1* KO male and oocyte activation in physiological conditions

**Figure 2:**
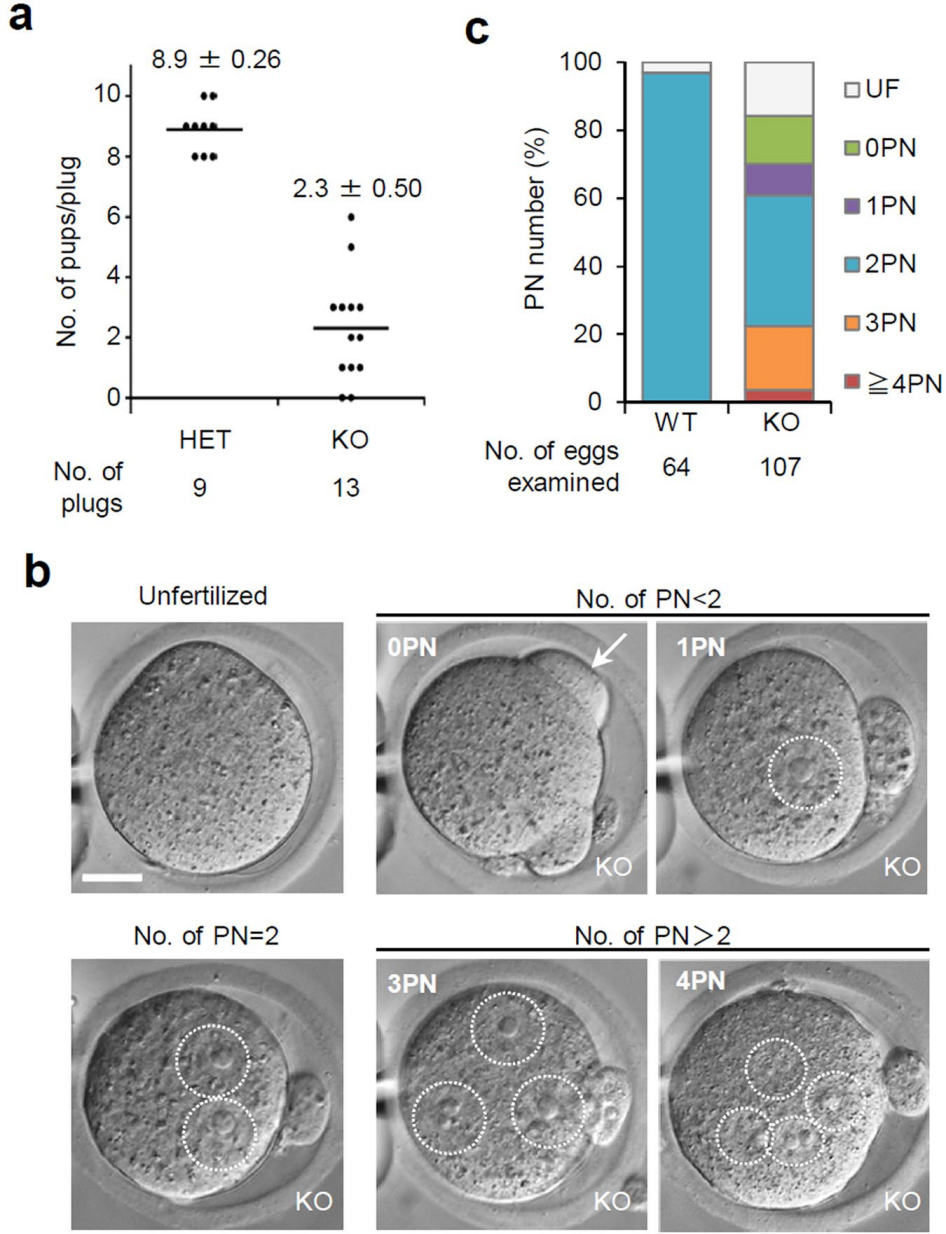
PLCζ1 is not essential for *in vivo* fertilization. (**a**) Fecundity of *Plcz1* heterozygous and KO males. The horizontal line and numerals indicate the mean numbers of pups/vaginal plug noted. (**b**) Oocytes collected from oviducts of females mated with *Plcz1* KO males at 10 h post coitus. Oocytes with various numbers of pronuclei were observed. Arrow and dotted circles represent the fertilizing cone and pronuclei, respectively. Scale bar = 20 μm. (**c**) The percentages of oocytes with the numbers of PN at 12 h post coitus.

The impact of PLCζ1 deficiency on male fertility was then examined by mating experiments with WT female mice. Copulations and vaginal plug formation were observed at normal rates for *Plcz1* KO males. Unexpectedly, *Plcz1* KO male mice sired healthy pups consistently, although the number of pups per copulation was significantly reduced (WT 8.9 pups vs. KO 2.3 pups; Fig. 2a). We then collected the oocytes from females after mating with WT or *Plcz1* KO males and examined fertilization status (Fig. 2b). With WT males, all oocytes showed monospermy with a distinct male PN at 12 h after coitus. With *Plcz1* KO males, more than 80% of oocytes (90/107) were fertilized, but fewer than one-half formed 2PN (41/90) (Fig. 2c). The remaining fertilized oocytes were observed to experience activation failure (no PN but with fertilization cone(s) at 12 h, indicated as 0PN; 15/90), abnormal activation (1PN; 10/90), or polyspermy (≥3PN; 24/90; Fig. 2c). When we recovered and cultured fertilized oocytes after mating with WT or KO males, increases in PN numbers were observed in some oocytes fertilized by KO sperm, implicating delays in fertilization and/or oocyte activation with *Plcz1* KO sperm (Supplementary Table 1). When we further cultivated 2PN oocytes and counted blastocysts at 108 h after coitus, preimplantation developmental ability was lower in oocytes fertilized by KO sperm than WT sperm (29/90 = 32.2% and 56/62 = 90.2%, respectively) (Supplementary Table 1). Thus, although *Plcz1* KO male mice are not sterile, the combined defects of activation failure and polyspermy may explain the reduced litter sizes, caused by male subfertility *in vivo*.

**Figure 3:**
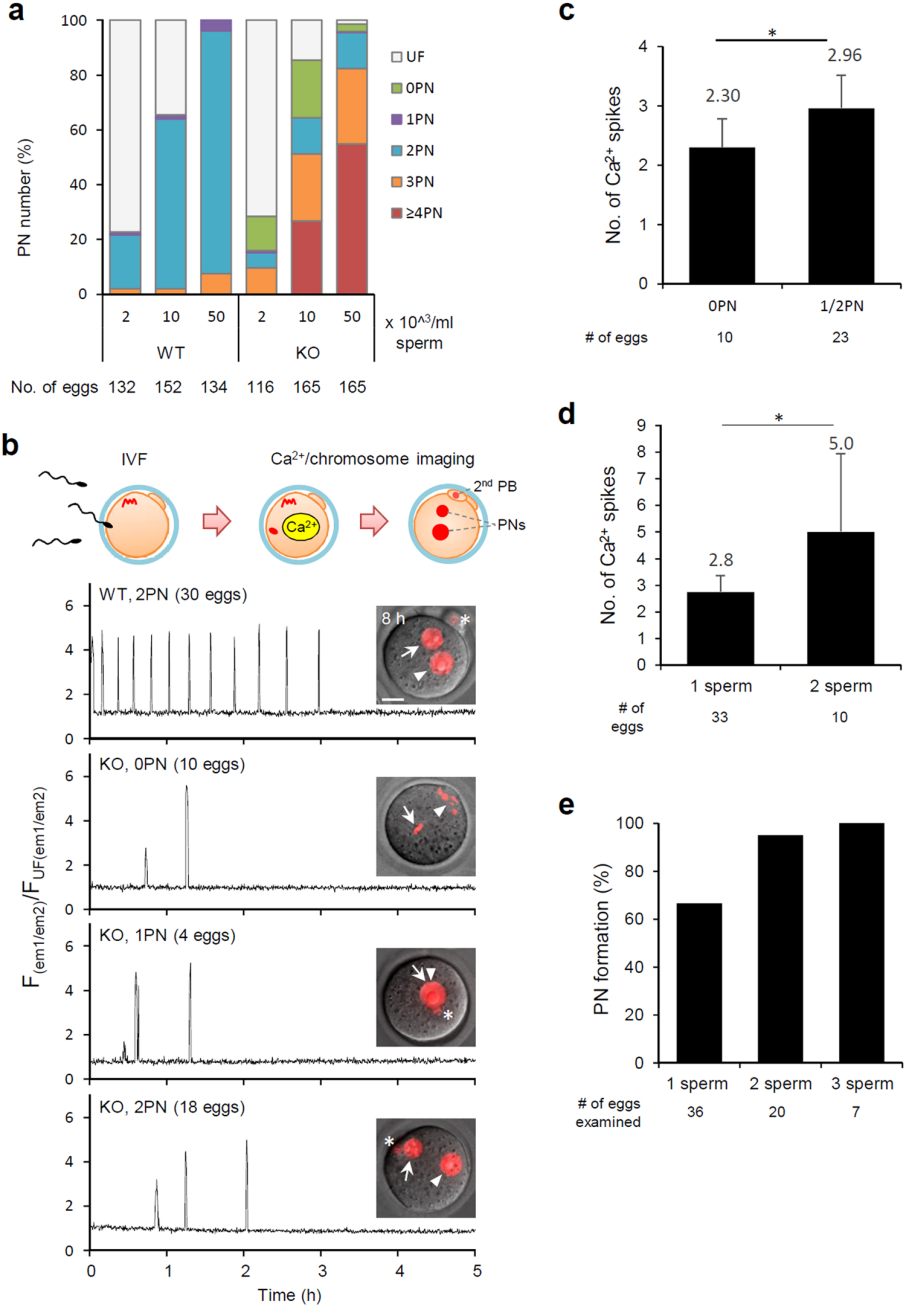
PLCζ1-independent oocyte activation causes abnormal fertilization. (**a**) The percentages of oocytes with each number of PN at 12 h after insemination. (**b**) Intracellular Ca^2+^ changes in oocytes after fertilization. Ratios of GEM-GECO fluorescence of oocytes with each genotype/number of pronuclei (representatives are shown). The numbers of PN formed (top left), and the mean numbers of Ca^2+^ spikes observed in oocytes (bottom right) are indicated. The numbers in parentheses indicate the numbers of oocytes observed. (**c**) Ca^2+^ spikes for oocytes fertilized by a single spermatozoon from *Plcz1* KO males. Numbers of Ca^2+^ spikes for each number of pronuclei are indicated. Oocytes with one or two PN contained significantly more spikes than 0PN oocytes (*P < 0.005). (**d**) Ca^2+^ spikes for oocytes fertilized with differing numbers of *Plcz1* KO sperm. Number of Ca^2+^ spikes for oocytes fertilized with 2 spermatozoa contained significantly more spikes than that fertilized with a single spermatozoon (*P < 0.005). (**e**) The percentages of oocytes that formed PN. Percentages are based on total numbers of oocytes fertilized with indicated number of *Plcz1* KO sperm.

To examine further the sperm fertilizing ability and oocyte activation ability, we performed *in vitro* fertilization (IVF) experiments at various sperm concentrations (2, 10, and 50 × 10^3^ spermatozoa/ml) (Fig. 3a, Supplementary Table 2). Fertilization rates were similar between WT and KO groups and increased along with sperm concentration. Characteristically, activation failure was evident with *Plcz1* KO spermatozoa at lower sperm concentrations (WT 0% vs. KO 12.4% at 2 × 10^5^/ml), and the polyspermy increased up to ~80% at higher sperm concentrations (WT 7.6% vs. KO 82.4% at 50 × 10^3^/ml; Fig. 3a). We next used live imaging to monitor intracellular Ca^2+^ concentrations. When we focused on monospermic fertilized oocytes, all the IVF fertilized oocytes with *Plcz1* KO spermatozoa demonstrated intracellular Ca^2+^ spikes regardless of PN formation (Fig. 3b, Supplementary Fig. 2c). The Ca^2+^ spike patterns were aberrant in these oocytes (usually with decreased amplitude), and the number of spikes significantly decreased (WT 12.0 ± 5.68 spikes, 30 eggs vs. KO 2.75 ± 0.65 spikes, 28 eggs, *P* < 0.05). When we analysed the oocytes fertilized by a single spermatozoon retrospectively, the number of spikes observed in the 0PN oocytes were significantly fewer than those in the oocytes with formed PNs (Fig. 3c), implicating the existence of a threshold of intracellular Ca^2+^ spikes to trigger PN formation. Consistently, as more spermatozoa fused, the number of Ca^2+^ spikes increased (Fig. 3d), and more oocytes resumed the cell cycle (Fig. 3e). These findings revealed that mammalian spermatozoa have an oocyte activation ability independent from PLCζ1. However, a single spermatozoon is insufficient and rarely triggers the resumption of the meiotic cell cycle, with incomplete oocyte activation leading to polyspermy.

### Polyspermy block systems in oocytes fertilized by KO spermatozoa

**Figure 4:**
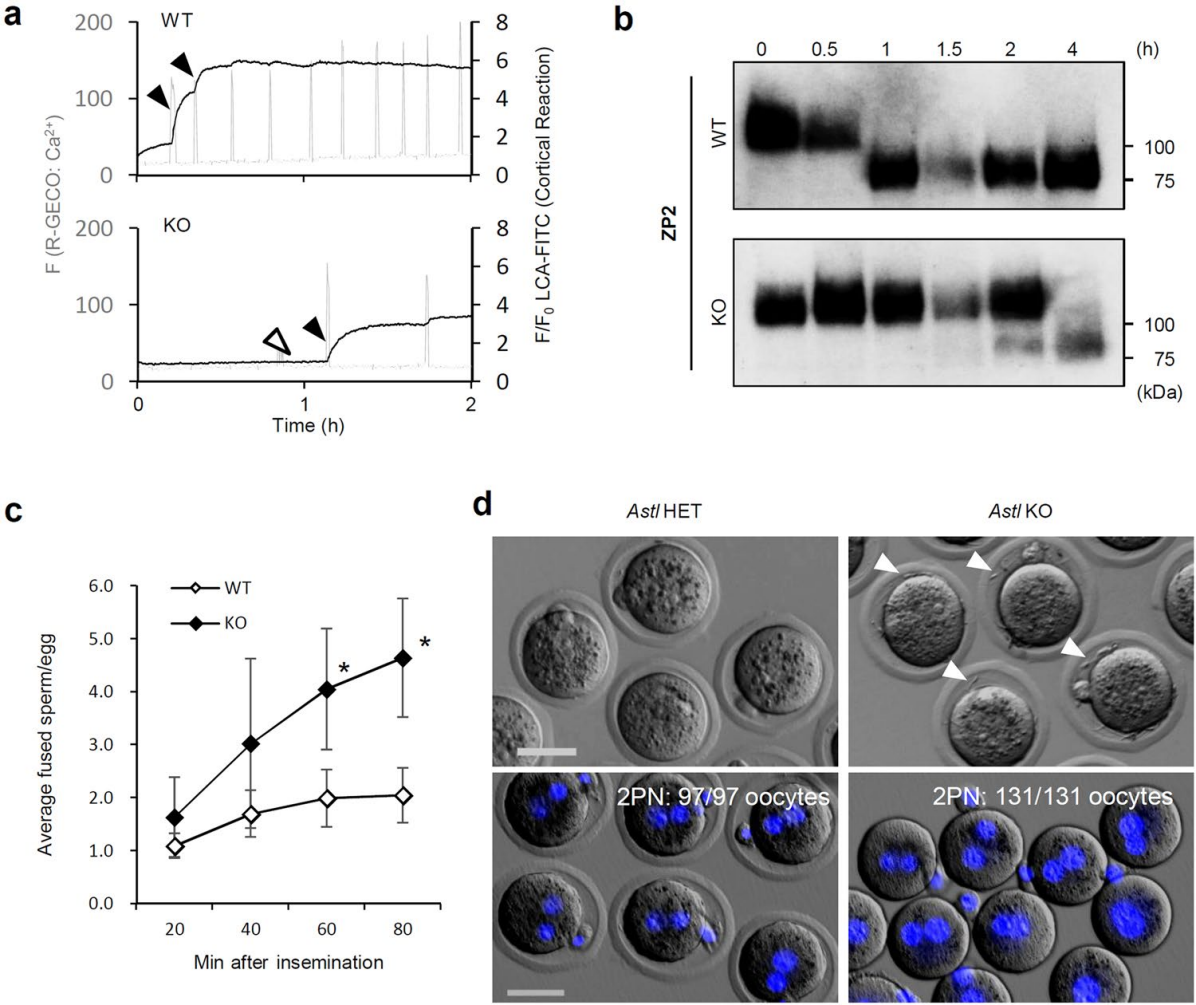
Delayed PMBP establishment by *Plcz1* deletion is a cause of polyspermy. (**a**) Representative fluorescence plots for R-GECO (intracellular Ca^2+^; gray) and LCA–FITC staining (CR; black) of oocytes after insemination. Black arrowheads highlight Ca^2+^ spikes that induced the CR, while white arrowheads indicate Ca^2+^ spikes that failed to initiate the CR. (**b**) ZP2 immunoblot of oocytes after insemination. Intact ZP2 and the cleaved C-terminal fragments of ZP2 were 120 kD and 90 kD, respectively. A full–length blot is presented in Supplementary Figure 7a. (**c**) PMBP establishment estimated with the number of fused sperm of *in vitro* fertilized oocytes. The data reflect the means of separate experiments for each genotype. (**d**) Oocytes collected from oviduct of *Astl* heterozygous or KO females mated with WT male. Bright field observation (upper) and nuclear staining (lower) using Hoechst 33342 (blue) at 12 h post coitum are indicated. Oocytes from *Astl* KO females were denuded to prevent spermatozoa within the *perivitelline* space (arrowheads) interfering with pronuclei counting. The numbers of 2PN oocytes/total oocytes from five females for each genotype are indicated. No KO oocyte exhibited polyspermy. Scale bar = 50 μm.

There are two major mechanisms to prevent polyspermy in mammals, the ZPBP and the PMBP. The process of the ZPBP is well documented. After fertilization, Ca^2+^ oscillations induce the cortical reaction (CR), an exocytosis event that releases the ASTL from the cortical granules into the extracellular space. This process exposes glycosylated proteins on the cell surface and can be visualized using *Lens culinaris* agglutinin-fluorescein isothiocyanate complex (LCA–FITC) labelling. The released ASTL cleaves the ZP2 protein and changes the ZP structure to prevent excess sperm entry^16^. When we visualized intracellular Ca^2+^ and the CR by LCA–FITC labelling^17^, a delayed and incomplete CR was observed coincident with a delayed Ca^2+^ spike by ~1 h that also had a lower amplitude in oocytes fertilized with *Plcz1* KO spermatozoa (Fig. 4a). Some oocytes failed to induce the CR during the initial Ca^2+^ spike. Immunoblotting indicated that ZP2 cleavage was completed by 4 h after insemination with *Plcz1* KO spermatozoa, whereas it was completed in 1 h with WT spermatozoa (Fig. 4b).

Ca^2+^ is also required for the PMBP, but its mechanism remains unknown^18^. Here, we assessed the establishment of PMBP by counting the number of spermatozoa fused with ZP-free oocytes (fusion index) at several time points. While the fusion index with WT spermatozoa plateaued at ~2 sperm/oocyte 60 min after insemination as reported previously^19^ (2.0 ± 0.54 at 60 min and 2.0 ± 0.51 at 80 min, respectively), the fusion indexes with *Plcz1* KO spermatozoa were significantly higher than with the WT and continued to increase even after 60 min (4.1 ± 1.14 at 60 min and 4.6 ± 1.12 at 80 min, respectively; Fig. 4c). We also examined PMBP status 12–15 h after the 1^st^ insemination. Oocytes fertilized with WT spermatozoa or with *Plcz1* KO spermatozoa rarely fused with WT spermatozoa at the 2^nd^ insemination (Supplementary Fig. 3a, b). Therefore, we conclude that both the ZPBP and PMBP were delayed but eventually established in the oocytes fertilized with PLCζ1-deficient spermatozoa.

The significance of these polyspermy blocking mechanisms was further challenged using *Astl* KO mice, which lacks the ZP2 cleavage. When *Astl* KO female mice were mated with WT male mice, the ZP2 protein remained intact after fertilization (Supplementary Fig. 3c), and extra spermatozoa accumulated in the perivitelline space indicating failure of the ZPBP, but the PMBP was still functional and all the oocytes exhibited 2PN (Fig. 4d). This maintenance of monospermic fertilization strongly indicates that the PMBP alone is sufficient to block polyspermy *in vivo*. Delayed PMBP is the most important reason for the polyspermy found with *Plcz1* KO male mice.

### Rescue of the infertility of *Plcz1* mutants by PLCζ1 supplementation

**Figure 5:**
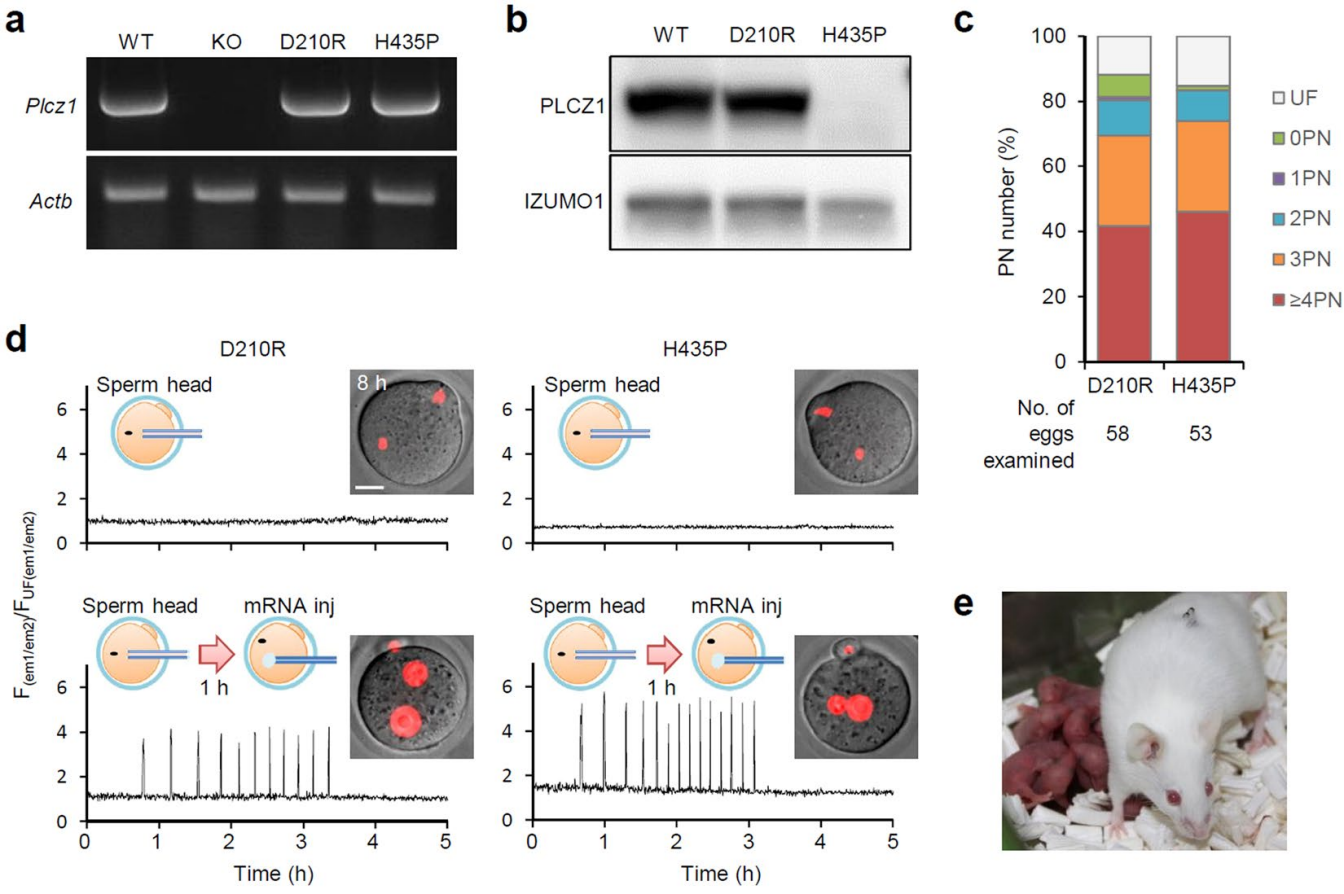
*Plcz1* mRNA can activate oocytes fertilized with *Plcz1* mutant spermatozoa. (**a**) RT–PCR analysis of testicular RNA. Actb; actin beta. (**b**) Immunoblot of sperm proteins. Full–length blots are presented in Supplementary Figure 5a and e. (**c**) The percentages of oocytes with specified numbers of PN 12 h after IVF with *Plcz1* point mutant mice. (**d**) Ratio of GEM-GECO fluorescence of oocytes after ICSI (upper) or 2 ng/µl mouse *Plcz1* mRNA injection following ICSI (lower). Images of oocytes 8 h after ICSI or mRNA injection are shown. Nuclei/PN were visualized with H2B-mCherry (red). Scale bar = 20 μm. (**e**) Representative host mother and pups from ICSI-rescued embryos.

Human genetic diseases are mainly caused by small mutations rather than gene deletions. There are several *PLCZ1* point mutations reported in infertile men^20,21^. Also, several artificial mutations were shown to influence oocyte activation ability as determined by injecting specific mRNAs into mouse oocytes^22^ (Supplementary Fig. 4a, b). Here, we phenocopied the human infertility-associated mutations in mice and introduced H435P (an equivalent mutation of human H398P) as well as D210R (an enzymatically dead mutation)^10^ mutations into mice using CRISPR/Cas9 technology (Supplementary Fig. 4c–e). Both mutant mRNAs were transcribed at WT levels in mutant mouse testes (Fig. 5a). Interestingly, when sperm proteins were analysed by immunoblotting, while the D210R protein was detected at ~74 kDa in similar amounts to WT mice, no H435P protein was detected at ~74 kDa (all 5 males examined for each genotype; Fig. 5b, Supplementary Fig. 5a). It should be noted that we occasionally observed ~20 kDa signal only in H435P sperm (3/5 males; Supplementary Fig. 5a), implicating the instability of H435P protein *in vivo*. Homozygous male mice carrying D210R or H435P mutations phenocopied *Plcz1* KO male mice in spermatogenesis, fertility, IVF, and ICSI outcomes (Fig. 5c,d, Supplementary Fig. 5b–d). By using these point mutant mice as male infertility models, we complemented oocyte activation with WT *Plcz1* mRNA injection. The optimized concentrations of human and mouse *Plcz1* mRNA for the activation of mouse oocytes^23^ were adopted in the study. When we injected mouse *Plcz1* mRNA (2 ng/μl × 1–3 pl) 1 h after ICSI using mutant spermatozoa, Ca^2+^ oscillations were followed by successful 2PN formation (Fig. 5d). The injection of 0.2 ng/μl human *PLCZ1* mRNA also successfully activated oocytes. Healthy pups were obtained after transplantation of the embryos obtained with *Plcz1* mRNA injection (Fig. 5e, Table 1).

**Table 1:** Development of oocytes after ICSI rescue with sperm from *Plcz1* KO or point mutant mice.

Sperm	Supplemented mRNA (conc.)	No. of eggs injected	No. of 2-cell stage embryos	No. of 2cell embryos transferred	Pregnant/Recipients	No. of pups born
WT	None	72	69	57	3/3	21
KO	mouse *Plcz1* (2 ng/µl)	78	78	62	3/3	31
D210R	mouse *Plcz1* (2 ng/µl)	73	71	49	2/3	13
H435P	mouse *Plcz1* (2 ng/µl)	73	70	45	3/3	17
KO	human *PLCz1* (0.2 ng/µl)	51	51	29	2/2	9

All offspring from *Plcz1* KO, D210R, or H435P spermatozoa were confirmed to possess the *Plcz1* KO, D210R, H435P allele by PCR.

## Discussion

Here, we have demonstrated that PLCζ1 is the long-sought SOAF in mice as indicated by the complete deletion of Ca^2+^ spikes in ICSI (Fig. 1). Further, we found that the existence of a PLCζ1-independent oocyte activation mechanism through normal fertilization where sperm–oocyte membrane interaction occurs. The activity is weak and barely triggers meiotic cell cycle resumption. Thus, this sperm–oocyte membrane interaction is probably a primitive but intrinsic mechanism for oocyte activation.

The results contrast with previous work reported at a conference in which *Plcz1* KO male mice were presented to be sterile with defective spermatogenesis^13^. However, this was likely to be an artifact of the KO strategy that produced a truncated product. Very recently, Hachem *et al*., reported a similar phenotype of *Plcz1* knockout mice^14^, but there is a notable difference. While we observed atypical but clear Ca^2+^ oscillations in all the oocytes after normal fertilization, Hachem *et al*., only found a single spike in one out of forty oocytes. In the present study, by labelling fused sperm chromosomes with Histon H2B-mCherry (Fig. 3b), we only analysed fertilized oocytes. Although the fertilization rate was not indicated, incorporation of unfertilized oocytes in the denominator may explain the difference. The correlation of Ca^2+^ spikes with the number of fused spermatozoa suggested that spermatozoa have a PLCζ1-independent Ca^2+^ spike inducing ability (Fig. 3c–e).

In the previous studies with electropermeabilization, more than four Ca^2+^ spikes were required to induce successful CR and subsequent cell cycle resumption^24,25^. However, we recently reported that CR completed within a few Ca^2+^ spikes during normal fertilization^17^. In this study, we found a significant difference in the number of Ca^2+^ spikes observed in (0PN) and activated (1 or 2PN) oocytes (2.30 ± 0.48 vs. 2.96 ± 0.56) with *Plcz1* KO sperm. Further analysis will be required, however, our data implicates that the mouse oocyte requires an average of three Ca^2+^ spikes to be activated.

There are several mechanisms proposed for Ca^2+^ dependent oocyte activation through sperm–oocyte interaction^26^, such as the Ca^2+^ conduit model found in *Caenorhabditis elegans*^27^, and a membrane receptor model found in *Xenopus*^28^. Further study with *Plcz1* KO mice will help to elucidate which mechanism(s) are used in mammals. It is interesting to note that newts, birds and insects are known to require polyspermy to ensure oocyte activation but use only a single decondensed sperm head to drive further development and ensure the correct diploid chromosome set in embryogenesis. However, polyspermy compromises further development in mammals. Here, we showed that PLCζ1 plays critical roles in both ZPBP and the PMBP. This ensures monospermic fertilization with successful oocyte activation and the prevention of extra spermatozoa from entering the ooplasm. We also showed that PMBP, rather than ZPBP, plays critical roles in the monospermic fertilization *in vivo* in mice. This finding will shed light onto development and the roles of these two mechanisms in various organisms.

In infertility clinics, because only a few oocytes are collected during retrieval, conventional IVF protocols have largely been replaced with ICSI protocols to increase the success rate and decrease the superovulatory burden on women^29^. Although ICSI ensures monospermic fertilization, oocyte activation failures still occur and have been reported as a cause of unexplained infertility^30^. As we observed polyspermy in *Plcz1* mutant mice, such patients might have polyspermy *in vivo* as well as oocyte activation failure following ICSI. If it is caused by male infertility, this form could possibly be treated by a combination of ICSI and PLCζ1 complementation *in vitro*.

## Methods

### Animals

Wild-type mice (C57BL/6 N × DBA/2) F1, also known as B6D2F1, were purchased from CLEA Japan (Tokyo, Japan) or Japan SLC (Shizuoka, Japan). All animal experiments were approved by the Animal Care and Use Committee of the Research Institute for Microbial Diseases, Osaka University, under ethics approval number Biken-AP-H25-02-0, and were performed in accordance with the relevant guidelines and regulations.

### Generation of *Plcz1* knockout mice with the CRISPR/Cas9 system

Two pX330 plasmids expressing single guide (sg)RNAs targeting the 5′ and 3′ regions of *Plcz1* (5′–TAGACGAAGAGCCCTCTATG–3′ and 5′–GTGCGAACCTTGAACCTTCC–3′) were co-transfected with human codon-optimized CAS9 to remove the coding region of *Plcz1* in EGR-G101 (C57BL/6) ES cells^31^. Embryonic stem (ES) cell clones were selected with puromycin. Correctly targeted ES cell clones and germ-line transmission were determined via polymerase chain reaction (PCR) using primers for the KO allele (primer c: 5′–GACCACATCTTTCATGTCC–3′ and primer d: 5′–AGCAACTGAGAATGCAACCC–3′) or the wild-type (WT) allele (primer a: 5′–ATGACTAGGGAGGAGCAGAGAC–3′ and primer b: 5′–ATTCCCATGACCACTCACTACC–3′). A founder mouse with a 50.8 kbp deletion was used to expand the colony.

### Intracytoplasmic sperm injection (ICSI)

ICSI was performed as described^32^. In brief, each sperm head was separated from the tail by applying a few piezo pulses, then injected into a denuded MII oocyte using a piezo manipulator (PrimeTech, Ibaraki, Japan). Whole sperm injection was carried out after immobilization of motile spermatozoa by a piezo pulse.

### Fertility testing

Sexually mature male mice of each genotype were caged with B6D2F1 sexually mature female mice. Copulation was confirmed by checking for vaginal plugs every morning and the numbers of pups were counted after caesarean section at 17.5 days post coitum.

### *In vivo* fertilization assay

WT female mice were injected with pregnant mare serum gonadotropin and human chorionic gonadotropin (hCG) at 48 hours intervals. Twelve hours after hCG injection, females were caged with control or mutant male mice. The female mice that copulated with males within 60 min were used in the study. Oocytes were collected from oviducts 7–8 h after coitus with male mice of each genotype and incubated in potassium-supplemented simplex optimized medium (KSOM) medium. The numbers of pronuclei were counted at 12 and 15 h post coitum. Subsequent embryonic development was observed to the blastocyst stage. For the analysis of *Astl* KO oocytes, heterozygous or homozygous KO female mice^17^ were superovulated, mated with WT males, and oocytes were collected as described above. To count the number of pronuclei, oocytes were stained with Hoechst 33342 (1 μg/ml, for 10 min) at 12 h after coitus. Zona removal with collagenase (Wako, Osaka, Japan) treatment at 100 μg/ml for 10 min was carried out for KO oocytes to remove sperm fluorescent signals in the perivitelline space.

### *In vitro* fertilization (IVF)

IVF was performed as described^33^. In brief, mature oocytes were collected and placed in 100 μl of Toyoda, Yokoyama, and Hoshi (TYH) medium. Spermatozoa were collected from the epididymides of sexually mature male mice of each genotype, and incubated in TYH medium for 2 h for capacitation. Capacitated sperm were added to the drop containing oocytes at a final concentration of 2–50 × 10^3^ sperm/ml. After 5 h of co-incubation, oocytes were washed and transferred to KSOM medium.

### Sperm motility analysis

Sperm motility was analysed as described^34^. Cauda epididymal spermatozoa were suspended in TYH medium^35,36^. Sperm motility was measured using the CEROS sperm analysis system (software version 12.3; Hamilton Thorne Biosciences, Beverly, MA, USA) at 30 min and 3 h after incubation.

### Analysis of acrosome reaction

Acrosomal exocytosis was analysed as described^36^. Spermatozoa expressing enhanced green fluorescent protein (EGFP) in the acrosome were suspended and incubated in TYH medium. At 30 min and 3 h after incubation, an aliquot of the suspension was stained with propidium iodide (final 10 μg/ml) and subjected to fluorescence-activated cell sorting with an EC800 cell analyzer (Sony, Tokyo, Japan). The viability and acrosomal integrity of sperm were determined by propidium iodide staining and the presence of acrosomal EGFP, respectively. A 525 nm and a 595 nm band path filter were used for EGFP and propidium iodide, respectively.

### Nuclear/pronuclear imaging

MII oocytes were subjected to microinjection in 4-(2-hydroxyethyl)-1-piperazineethanesulfonic acid Chatot, Ziomek, Bavister (Hepes-CZB) medium with mRNA for histone H2B-mCherry (5 ng/μL) (a kind gift from Dr. Kazuo Yamagata from Kindai University, Wakayama, Japan)^37^, followed by incubation in fresh KSOM medium at 37 °C under 5% CO_2_ in humidified air for 3 h. After ICSI, the oocytes were incubated for at least 10 min to allow membrane sealing, followed by quick transfer into glass-bottomed chambers for observation of pronuclear formation and embryo development using spinning-disk confocal microscopy. The second polar body was distinguishable from the first polar body by its positive mCherry signal. The formation of pronuclei was examined both by bright field observation and by the sized/shape of the mCherry fluorescence.

### Ca^2+^ imaging

Ca^2+^ imaging was performed as described^17^. Metaphase (M) stage II oocytes were subjected to microinjection with a mixture of mRNAs for GEM-GECO (60 ng μl) and histone H2B-mCherry (5 ng/μl) in Hepes-CZB medium, followed by incubation in fresh KSOM medium at 37 °C under 5% CO_2_ in humidified air for 3 h. ICSI was performed as described above within 5 min. IVF was performed as described^17^ within 10 min using oocytes subjected to partial zona dissection. After fertilization by either method, the oocytes were quickly transferred into glass-bottomed chambers for observation using spinning-disk confocal microscopy. Images of Ca^2+^ dynamics were taken at 20-s intervals for 5 h. After Ca^2+^ imaging, images of nuclei/pronuclei with H2B-mCherry were acquired at 15-min intervals for 24 h for evaluation of the number of pronuclei and fused sperm.

### Imaging of the cortical reaction

Oocytes were observed as described^17^. Oocytes microinjected with R-GECO (30 ng/μl) were inseminated with capacitated spermatozoa at a concentration of 1.0 × 10^6^/ml for 10 min. They were observed in CZB medium containing *Lens culinaris* agglutinin-fluorescein isothiocyanate (LCA–FITC) (5 ng/μl) (J-Oil Mills, Tokyo, Japan).

### Zone pellucida protein (ZP2) cleavage assay

ZP2 cleavage assay was performed as described^17^. Oocytes were collected 0.5, 1, 1.5, 2, 3, and 4 h after IVF and lysed in 3× Tris-glycine sodium dodecyl sulfate (SDS) loading buffer, separated on 5–20% Tris-glycine gels by SDS–polyacrylamide gel electrophoresis (PAGE), transferred to polyvinylidene fluoride membranes (Invitrogen Life Technologies, Carlsbad, CA, USA), blocked in 7.5% nonfat milk in Tris–Bufferd Saline containing 0.05% Tween 20, and probed with primary antibodies for ZP2 (gift from Dr. Jurrien Dean of the National Institute of Diabetes and Digestive and Kidney Diseases, Bethesda, MD, USA), followed by secondary antibodies conjugated with horseradish peroxidase^38^. Chemiluminescence was performed with ECL Plus (GE Healthcare, Little Chalfont, UK), and signals were acquired with the Luminescent Image Analyzer LAS-3000 (Fujifilm, Tokyo, Japan).

### Plasma membrane block to polyspermy (PMBP) assay

The PMBP assay was performed using two methods. In the first method, the fusion index was counted as described previously^39^. The ZP of denuded MII oocytes were removed by incubation in TYH medium containing 100 μg/ml collagenase (Type I-S, Merck KGaA, Darmstadt, Germany) for 5 min. ZP-free oocytes were preloaded with 1 μg/ml Hoechst 33342 for 10 min and washed four times. After 20, 40, 60, or 80 min of insemination with WT or KO spermatozoa at a concentration of 0.5 × 10^5^/ml, the oocytes were fixed in 0.25% glutaraldehyde and observed. Only the fused sperm heads were detected as fluorescent positive and counted. In the second method, oocytes were fertilized by conventional IVF with WT or PLCζ1-deficient spermatozoa at a concentration of 0.5 × 10^5^/ml, as described above. After 12–15 h of incubation in KSOM medium,the ZP of fertilized oocytes were removed by treatment with acidic Tyrode’s solution^40^ followed by staining with Hoechst 33342 for 10 min and four washes. After 30 min of insemination using WT sperm at a concentration of 0.5 × 10^5^/ml, the oocytes were fixed with 0.25% glutaraldehyde and observed. In this method, all the nuclei are stained and detected as fluorescent positive, thus fused and non-fused sperm nuclei were distinguished by size and shape.

### Antibodies

Rabbit polyclonal antibody was produced by immunization with mouse PLCz1 polypeptide (GYRRVPLFSKSGANLEPSS)^10^. The monoclonal antibody against IZUMO1 (KS64–125) was obtained as described^41^. The anti-ZP2 antibody used in immunoblotting analysis was a gift from Dr. Jurrien Dean of the National Institute of Diabetes and Digestive and Kidney Diseases.

### Data Availability

Mutant *Plcz1* mice used in this study are available through Riken BioResource Center, Japan (http://en.brc.riken.jp/). The stock numbers (RBRC numbers) for B6D2-Plcz1 <em1Osb>, B6D2-Plcz1 <em3(D210R)Osb>, and B6D2;B6-Plcz1 <em4(H435P)Osb> are 10014, 10093, and 09988, respectively. All other data are available from the authors on reasonable request.

## Electronic supplementary material

Supplementary Information

## References

[1] Cuthbertson KS, Cobbold PH (1985). Phorbol ester and sperm activate mouse oocytes by inducing sustained oscillations in cell Ca2+. Nature.

[2] Ducibella T, Fissore R (2008). The roles of Ca2+, downstream protein kinases, and oscillatory signaling in regulating fertilization and the activation of development. Dev Biol.

[3] Swann K (1990). A cytosolic sperm factor stimulates repetitive calcium increases and mimics fertilization in hamster eggs. Development.

[4] Stice SL, Robl JM (1990). Activation of mammalian oocytes by a factor obtained from rabbit sperm. Mol Reprod Dev.

[5] Wu AT (2007). PAWP, a sperm-specific WW domain-binding protein, promotes meiotic resumption and pronuclear development during fertilization. J Biol Chem.

[6] Parrington J, Swann K, Shevchenko VI, Sesay AK, Lai FA (1996). Calcium oscillations in mammalian eggs triggered by a soluble sperm protein. Nature.

[7] Nomikos M. *et al*. Functional disparity between human PAWP and PLCzeta in the generation of Ca^2+^ oscillations for oocyte activation. *Mol Hum Reprod* (2015). 10.1093/molehr/gav034 26116451

[8] Wolny YM (1999). Human glucosamine-6-phosphate isomerase, a homologue of hamster oscillin, does not appear to be involved in Ca2+ release in mammalian oocytes. Mol Reprod Dev.

[9] Satouh Y, Nozawa K, Ikawa M (2015). Sperm Postacrosomal WW Domain-Binding Protein Is Not Required for Mouse Egg Activation. Biol Reprod.

[10] Saunders CM (2002). PLC zeta: a sperm-specific trigger of Ca(2+) oscillations in eggs and embryo development. Development.

[11] Putney JW, Tomita T (2012). Phospholipase C signaling and calcium influx. Adv Biol Regul.

[12] Kouchi Z (2004). Recombinant phospholipase Czeta has high Ca2+ sensitivity and induces Ca2+ oscillations in mouse eggs. J Biol Chem.

[13] Ito, M. *et al*. Arrest of spermatogenesis at round spermatids in PLCZ1-deficient mice. *11th International symposium on Spermatology* (abstract) (2010).

[14] Hachem, A. *et al*. PLCzeta is the physiological trigger of the Ca^2+^ oscillations that induce embryogenesis in mammals but offspring can be conceived in its absence. *Development* (2017). 10.1242/dev.150227 PMC5592814 28694258

[15] Weissmann C, Flechsig E (2003). PrP knock-out and PrP transgenic mice in prion research. Br Med Bull.

[16] Burkart AD, Xiong B, Baibakov B, Jimenez-Movilla M, Dean J (2012). Ovastacin, a cortical granule protease, cleaves ZP2 in the zona pellucida to prevent polyspermy. J Cell Biol.

[17] Satouh Y, Nozawa K, Yamagata K, Fujimoto T, Ikawa M (2017). Viable offspring after imaging of Ca^2+^ oscillations and visualization of the cortical reaction in mouse eggs. Biol Reprod.

[18] McAvey BA, Wortzman GB, Williams CJ, Evans JP (2002). Involvement of calcium signaling and the actin cytoskeleton in the membrane block to polyspermy in mouse eggs. Biol Reprod.

[19] Gardner AJ, Evans JP (2006). Mammalian membrane block to polyspermy: new insights into how mammalian eggs prevent fertilisation by multiple sperm. Reprod Fertil Dev.

[20] Heytens E (2009). Reduced amounts and abnormal forms of phospholipase C zeta (PLCzeta) in spermatozoa from infertile men. Hum Reprod.

[21] Yoon SY (2008). Human sperm devoid of PLC, zeta 1 fail to induce Ca(2+) release and are unable to initiate the first step of embryo development. J Clin Invest.

[22] Nomikos M (2011). Male infertility-linked point mutation disrupts the Ca2+ oscillation-inducing and PIP(2) hydrolysis activity of sperm PLCzeta. Biochem J.

[23] Cox LJ (2002). Sperm phospholipase Czeta from humans and cynomolgus monkeys triggers Ca2+ oscillations, activation and development of mouse oocytes. Reproduction.

[24] Ducibella T (2002). Egg-to-embryo transition is driven by differential responses to Ca(2+) oscillation number. Dev Biol.

[25] Ducibella T, Schultz RM, Ozil JP (2006). Role of calcium signals in early development. Semin Cell Dev Biol.

[26] Miyazaki s (2006). Thirty years of calcium signals at fertilization. Semin Cell Dev Biol.

[27] Takayama J, Onami S (2016). The Sperm TRP-3 Channel Mediates the Onset of a Ca(2+) Wave in theFertilized C. elegans Oocyte. Cell Rep.

[28] Sato K, Tokmakov AA, Iwasaki T, Fukami Y (2000). Tyrosine kinase-dependent activation of phospholipase Cgamma is required for calcium transient in Xenopus egg fertilization. Dev Biol.

[29] Johnson LN, Sasson IE, Sammel MD, Dokras A (2013). Does intracytoplasmic sperm injection improve the fertilization rate and decrease the total fertilization failure rate in couples with well-defined unexplained infertility? A systematic review and meta-analysis. Fertil Steril.

[30] Vanden Meerschaut F (2013). Diagnostic and prognostic value of calcium oscillatory pattern analysis for patients with ICSI fertilization failure. Hum Reprod.

[31] Oji A (2016). CRISPR/Cas9 mediated genome editing in ES cells and its application for chimeric analysis in mice. Sci Rep.

[32] Kimura Y, Yanagimachi R (1995). Mouse oocytes injected with testicular spermatozoa or round spermatids can develop into normal offspring. Development.

[33] Tokuhiro K, Ikawa M, Benham AM, Okabe M (2012). Protein disulfide isomerase homolog PDILT is required for quality control of sperm membrane protein ADAM3 and male fertility [corrected]. Proc Natl Acad Sci USA.

[34] Miyata H (2015). Sperm calcineurin inhibition prevents mouse fertility with implications for male contraceptive. Science.

[35] Toyoda Y, Yokoyama M, Hoshi T (1971). Studies on the fertilization of mouse egg *in vitro*. Jpn J Anim Reprod.

[36] Nakanishi T (1999). Real-time observation of acrosomal dispersal from mouse sperm using GFP as a marker protein. FEBS Lett.

[37] Yamagata K, Suetsugu R (2009). Wakayama T. Long-term, six-dimensional live-cell imaging for the mouse preimplantation embryo that does not affect full-term development. J Reprod Dev.

[38] Gahlay G, Gauthier L, Baibakov B, Epifano O, Dean J (2010). Gamete recognition in mice depends on the cleavage status of an egg’s zona pellucida protein. Science.

[39] Inoue N, Ikawa M, Isotani A, Okabe M (2005). The immunoglobulin superfamily protein Izumo is required for sperm to fuse with eggs. Nature.

[40] Maleszewski M, Kimura Y, Yanagimachi R (1996). Sperm membrane incorporation into oolemma contributes to the oolemma block to sperm penetration: evidence based on intracytoplasmic sperm injection experiments in the mouse. Mol Reprod Dev.

[41] Ikawa M (2011). Calsperin is a testis-specific chaperone required for sperm fertility. J Biol Chem.

