# Supplementary material for: Sperm-borne phospholipase C zeta-1 ensures monospermic fertilization in mice
Source: Sci Rep. 2018 Jan 22;8:1315. doi: 10.1038/s41598-018-19497-6 (PMC5778054; doi:10.1038/s41598-018-19497-6)
Supplement: Supplementary file 1 — Supplementary Information [file 41598_2018_19497_MOESM1_ESM.pdf]

Supplementary Information:

Sperm-borne phospholipase C zeta-1 ensures monospermic fertilization in mice

Kaori Nozawa[1,2,*], Yuhkoh Satouh[1,*], Takao Fujimoto[1,3], Asami Oji[1,3], Masahito Ikawa[1,2,3,4,§]

[1] Research Institute for Microbial Diseases, Osaka University, Suita, Osaka 5650871, Japan.

[2] Graduate School of Medicine, Osaka University, Suita, Osaka 5650871, Japan.

[3] Graduate School of Pharmaceutical Sciences, Osaka University, Suita, Osaka 5650871, Japan.

[4] The Institute of Medical Science, The University of Tokyo, Tokyo 1088639, Japan

* Contributed equally to this work.

§ To whom correspondence should be addressed.

The authors declare no competing financial interests.

Corresponding author:

Masahito Ikawa

Research Institute for Microbial Diseases, Osaka University, 3-1 Yamada-oka, Suita, Osaka 5650871 JAPAN

Phone: +81-6-6879-8375, Fax: +81-6-6879-8376, E-mail: ikawa@biken.osaka-u.ac.jp

Supplementary Figures

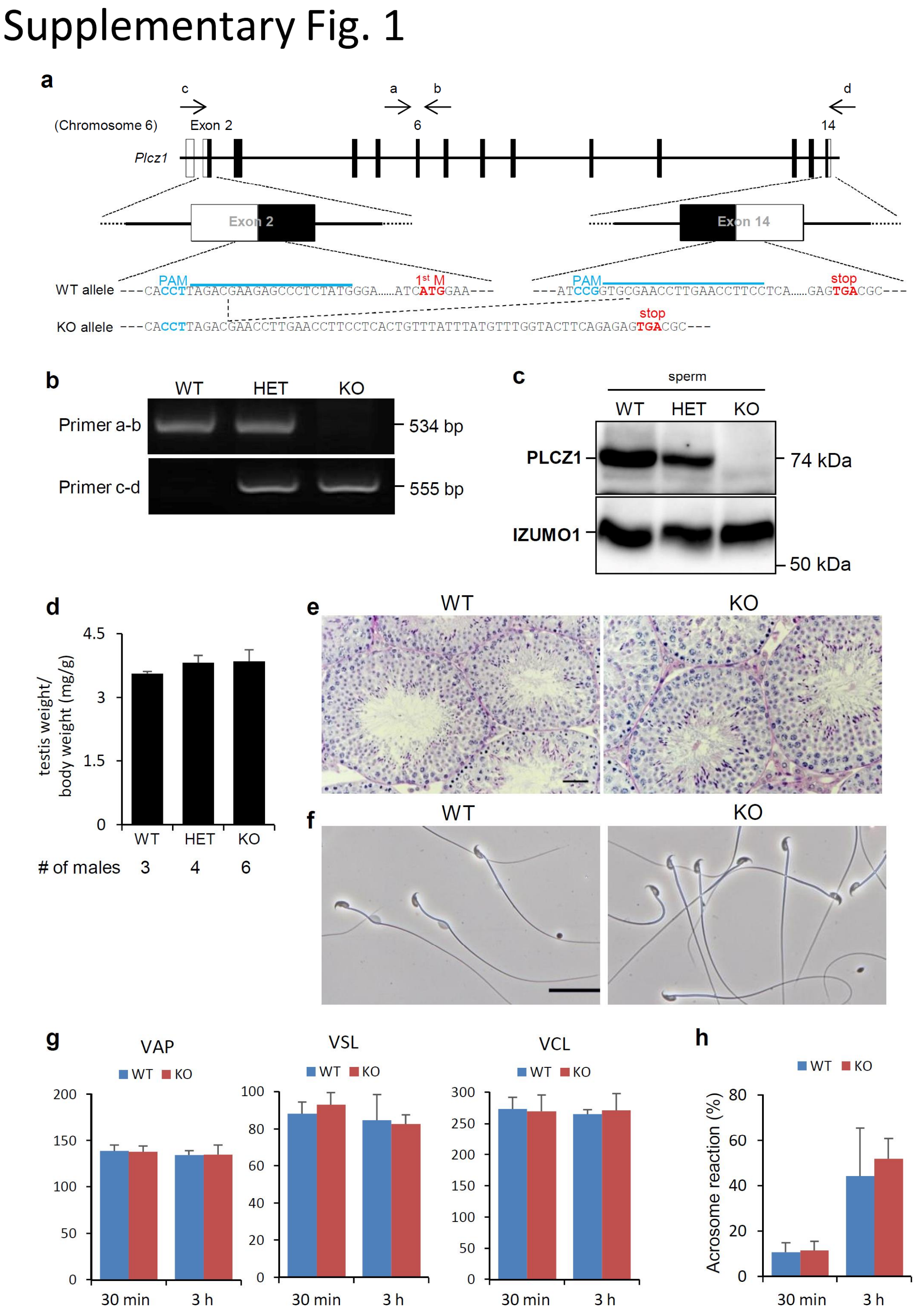

Supplementary Fig. 1 | Generation of *Plcz1* KO mice using CRISPR/Cas9.

a, Genomic structure of mouse *Plcz1*. White and black shadings indicate untranslated and coding regions, respectively. Sequences of nucleotides in exon 2 and exon 14 are shown. Blue letters indicate the PAM sequence and lines indicate sgRNAs that target regions near the start codon or stop codon (both in red). In total, 50,827 bp of the *Plcz1* gene was deleted. **b,** Genotyping of *Plcz1* alleles. Primers a and b amplify a 534 bp amplicon for the WT allele, and primers c and d amplify a 555 bp PCR product for the KO allele. **c,** Immunoblot analysis of cauda epididymal sperm lysates demonstrates absence of PLCζ1 in *Plcz1* KO mice. Fullï length blots are presented in Supplementary Figure 7b. (**d–f**). Testicular weight (**d**), testicular sections stained with periodic acid-Schiff (PAS)/haematoxylin (**e**), phase contrast images of cauda epididymal spermatozoa (**f**). Scale bars = 50 μm (**e**) and 20 μm (**f**). **g,** Sperm motility at 30 min and 3 h after sperm suspension. VAP: average path velocity, VSL: straight-line velocity, VCL: curvilinear velocity. **h,** The percentage of sperm losing acrosomal EGFP at 30 and 180 min after incubation in capacitation media was examined with fluorescence activated cell sorting.

Supplementary Fig. 2

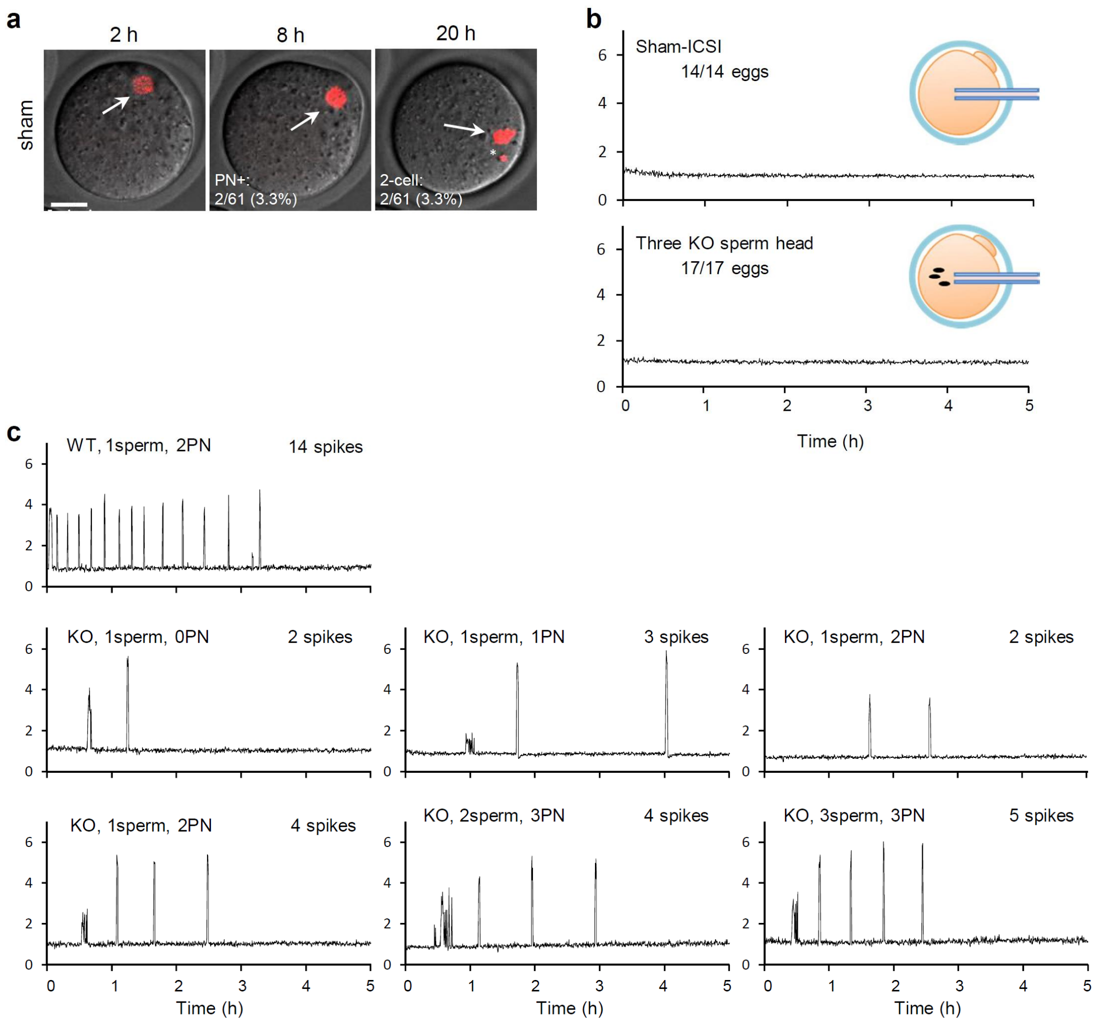

Supplementary Fig. 2 | Oocyte activation ability of *Plcz1* KO spermatozoa using ICSI or IVF.

a, Oocytes at 2, 8, and 20 h after injection of PVP medium. Oocytes injected with medium did not resume the meiotic cell cycle. Nuclei/PN were visualized with H2B-mCherry. Fractions indicate the numbers of oocytes/total that developed to the PN or 2-cell stage. Arrows and the white asterisk indicate chromosomes and fragmented chromosomes, respectively. Scale bar = 20 μm. **b,** Ratio of GEM-GECO fluorescence of oocytes after injection. Ca^{2+} oscillations were absent after sham ICSI and after ICSI with three heads of *Plcz1* KO spermatozoa. Fractions on tracings indicate the numbers of oocytes/total exhibiting the indicated patterns. **c,** Ca^{2+} changes in oocytes after insemination. The patterns not described in Fig. 3 are shown. The sperm genotype, the numbers of fertilized sperm per oocyte, and fate at PN stage of oocytes are indicated in the upper left of each graph. The number of Ca^{2+} spikes of each oocyte is indicated in the upper right.

Supplementary Fig. 3

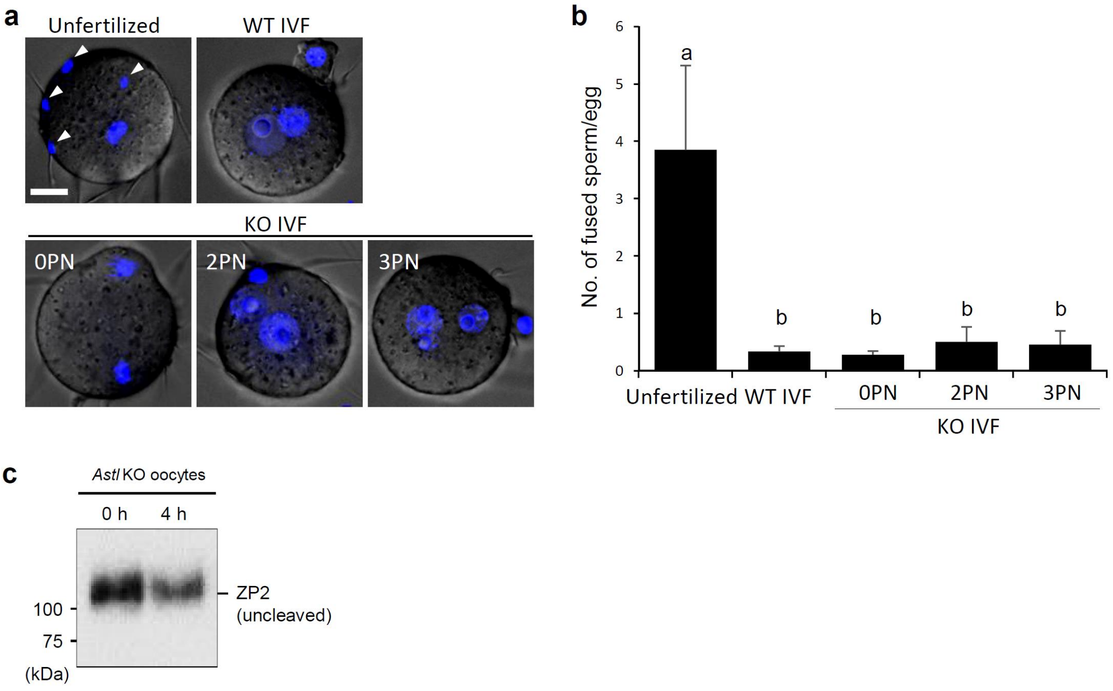

Supplementary Fig. 3 | Assessment of the plasma membrane block to polyspermy (PMBP) in oocytes fused with *Plcz1* KO spermatozoa and ZP2 cleavage in *Astl* KO oocytes.

a, Establishment of PMBP was examined at 12ï 15 h after insemination. Nuclei were visualized with Hoechst 33342. Arrowheads indicate nuclei of sperm fused at the second insemination (condensed). Scale bar = 20 μm. **b,** Mean numbers of sperm heads fused per oocytes at the second insemination. Significantly fewer numbers of sperm heads fused with oocytes after IVF with *Plcz1* KO spermatozoa (KO IVF) compared with unfertilized oocytes. Different letters on top of bars indicate significant difference ($P < 0.05$). **c,** Immunoblot of oocytes from *Astl* KO females at 0 or 4 h after insemination using anti-ZP2 antibodies. *Astl* KO oocytes did not cleave ZP2 at 4 h after insemination with WT sperm. Intact ZP2 and the cleaved C-terminal fragment of ZP2 measured 120 kD and 90 kD, respectively. Molecular mass is indicated at the right. See also Fig. 3d. A fullï length blot is presented in Supplementary Figure 7c.

Supplementary Fig. 4

a

mRNA	WT			D210R			H435P			S536L			S537L			H_2O
Concentration (ng/µl)	2	10	50	2	10	50	2	10	50	2	10	50	2	10	50	-
No. of oocytes used	46	87	73	48	80	45	46	95	43	43	34	27	46	30	24	84
M2	0	0	2	47	80	44	45	95	43	0	0	0	0	0	3	84
1PN	46	84	65	0	0	0	1	0	0	43	34	25	46	30	21	0
2PN	0	3	6	1	0	1	0	0	0	0	0	2	0	0	0	0
Activated oocytes (%)	100	100	97.3	2.1	0.0	2.2	2.2	0.0	0.0	100	100	100	100	100	87.5	0.0
# of experiments	2	4	3	2	4	2	2	4	2	2	2	1	2	2	1	4

mRNA	WT-EGFP		D210R-EGFP		H435P-EGFP		S536L-EGFP		S537L-EGFP		H_2O
Concentration (ng/µl)	2	50	2	50	2	50	2	50	2	50	-
No. of oocytes used	44	44	45	43	41	41	40	44	46	37	42
M2	1	0	45	43	41	41	0	0	0	0	40
1PN	43	44	0	0	0	0	40	44	46	37	1
2PN	0	0	0	0	0	0	0	0	0	0	1
Activated oocytes (%)	97.7	100	0.0	0.0	0.0	0.0	100	100	100	100	4.8
# of experiments	2	2	2	2	2	2	2	2	2	2	2

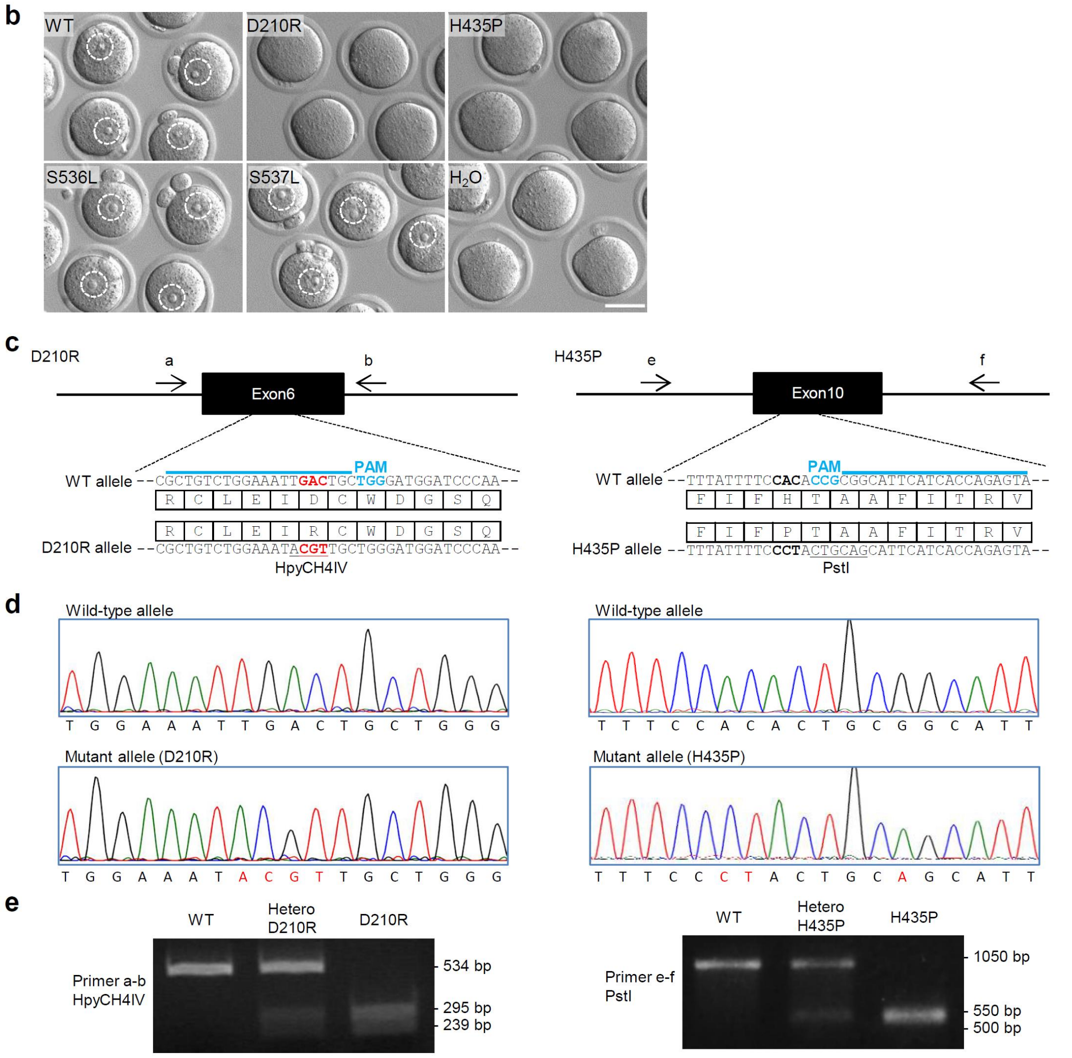

Supplementary Fig. 4 | Generation of *Plcz1* point mutant mice using CRISPR/Cas9.

a, Activation in oocytes after microinjection of mutant *Plcz1* mRNA. Oocytes were observed 8 h after mRNA injection. Expression of EGFP-tagged PLCζ1 protein was confirmed by green fluorescence. **b,** Oocytes 8 h

after microinjection of mRNA for WT mouse *Plcz1*, D210R, H435P, S536L or S537L point mutants of mouse *Plcz1*. Scale bar = 50 μm. Dotted circles indicate pronuclei. **c,** Generation of *Plcz1* D210R and H435P point mutant mice using the CRISPR/Cas9 system. Sequences of nucleotides and their corresponding amino acids for both WT and the mutated *Plcz1* allele are shown. Blue lines indicate sequence of sgRNAs and blue letters indicate PAM sequences. The codons in red are targeted for mutation. Restriction enzyme sites of HpyCH4VI and PstI are indicated by underlines. **d,** Chromatograms of the F2 generation (homozygous mice). Mutated sequences are shown in red. **e,** Genotyping of *Plcz1* point mutants. Primers a and b amplify a 534 bp PCR product. The PCR product from D210R mice was digested using HpyCh4IV. 1050 bp PCR product from H435P mutant allele using primer c and d was digested using PstI.

Supplementary Fig. 5

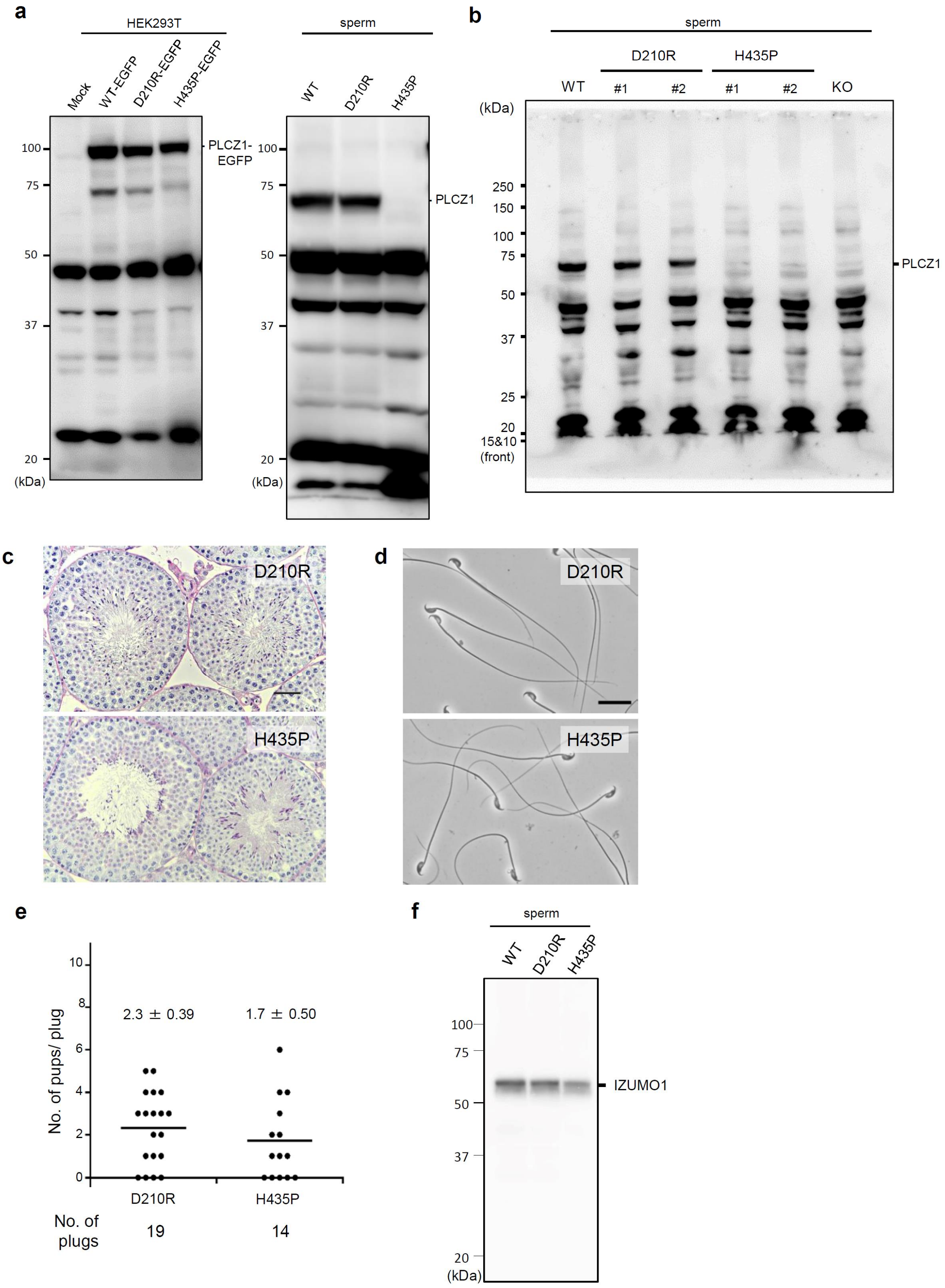

Supplementary Fig. 5 | Testis and sperm morphology, fecundity, and immunoblot of *Plcz1* point mutant mice.

a, (left) EGFP-tagged mouse PLCζ1 was overexpressed in HEK293T cells. D210R, H435P, and WT PLCζ1 were detected by immunoblotting using an anti-PLCζ1 antibody. (right) Fullï length immunoblot of sperm proteins from WT, D210R, and H435P mice using anti-PLCζ1 antibodies. H435P spermatozoa display an increased signal near the bottom of the lane. **b,** Fullï length immunoblot of sperm proteins from WT, D210R, H435P, and KO mice using an anti-PLCζ1 antibodies. Specimens were additionally collected from one WT male, two D210R males, two H435P males, and one KO male to examine the signal incidence at ~74 kDa and ~20 kDa. The signals at ~74 kDa in similar amounts to WT mice were detected among 5/5 examined D210R males in total. No signal was detected at ~74 kDa in 5/5 examined H435P males, whereas the signals at ~20 kDa was found to be detected occasionally (3/5 positive males in total). (**c, d**). Testicular sections stained with PAS and haematoxylin (**c**), cauda epididymal spermatozoa (**d**). Scale bars = 50 μm (**c**), and 20 μm (**d**). **e,** Number of pups from *Plcz1* point mutant males. Male *Plcz1* D210R and H435P mutant mice displayed subfertility. The mean numbers of pups/vaginal plug are indicated. **f,** Fullï length immunoblot of sperm proteins from WT, D210R, and H435P mice using anti-IZUMO1 antibody. A cropped image is indicated in Figure 4b.

Supplementary Fig. 6

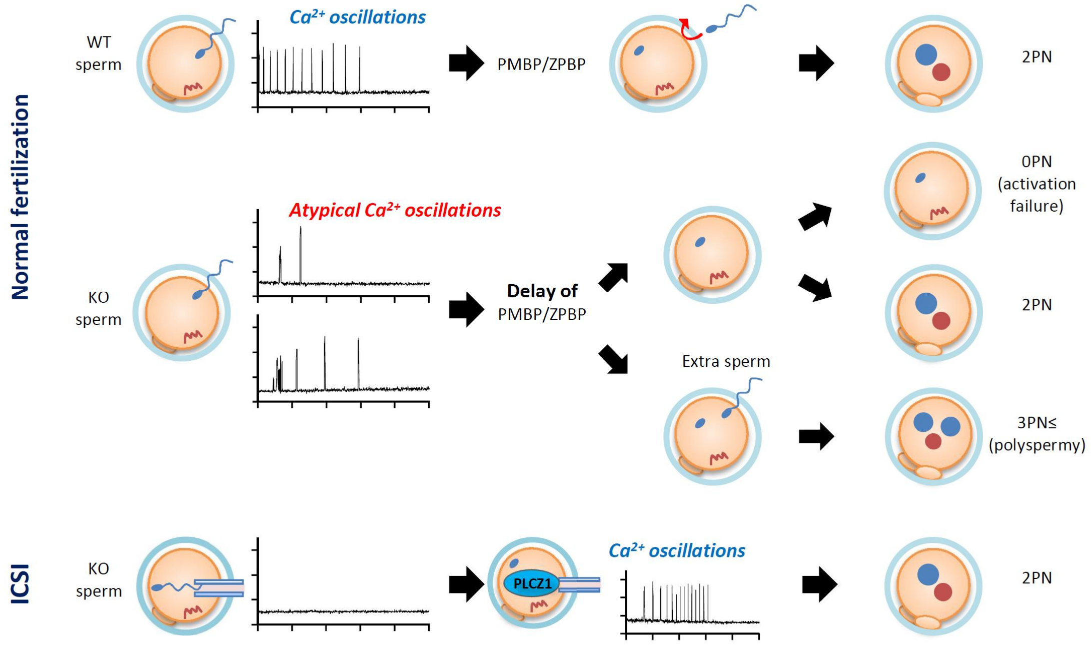

Supplementary Fig. 6 | Sperm-borne phospholipase C zeta-1 ensures monospermic fertilization in mice. A schematic drawing of this study. (upper) Normal fertilization and oocyte activation by WT or *Plcz1* KO sperm. (lower) ICSI of *Plcz1* KO sperm and supplementation with *Plcz1* mRNA.

Supplementary Fig. 7

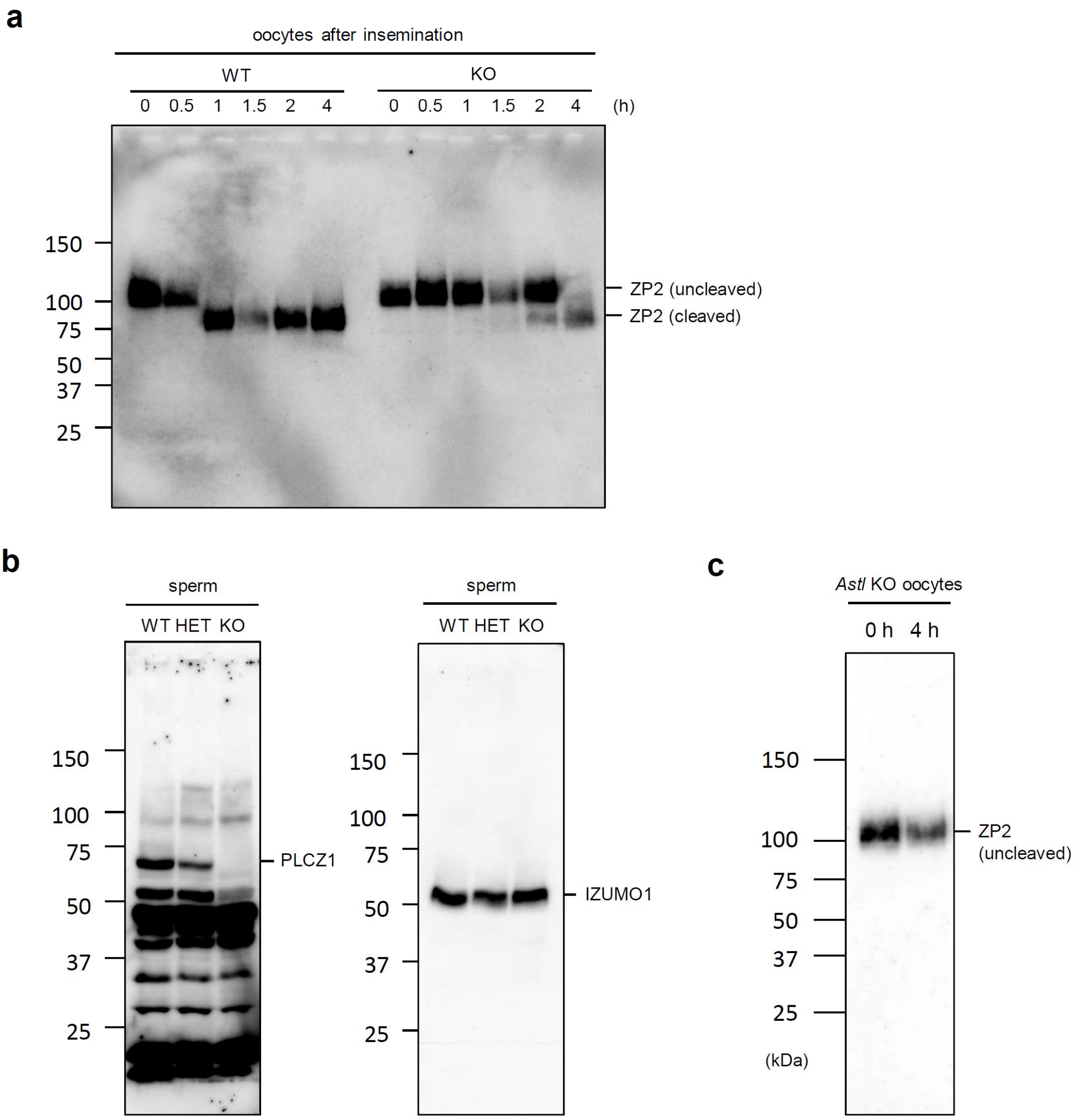

Supplementary Fig. 7 | Full-length blots.

a, ZP2 immunoblot of oocytes after insemination. Cropped images are indicated in Figure 3d. **b,** Immunoblot of cauda epididymal sperm lysates using an anti-PLCζ1 antibody (left) or anti-IZUMO1 antibody (right). Cropped images are indicated in Supplementary Figure 1c. **c,** ZP2 Immunoblot of oocytes from *Astl* KO females at 0 or 4 h after insemination. A cropped image is indicated in Supplementary Figure 3c.

Supplementary Tables

Supplementary Table 1. Development of oocytes collected after natural mating

WT

hpc 12 h		15 h		36 h (1.5 d)	108 h (4.5 d)
# of eggs		# of eggs		# of eggs developing to 2-cell stage	# of eggs developing to blastocyst
UF	2	UF	2	0	0
		0 PN	0	0	0
0 PN	0	0 PN	0	0	0
		1 PN	0	0	0
		2 PN	0	0	0
		3 PN	0	0	0
		≥4 PN	0	0	0
1 PN	0	1 PN	0	0	0
		2 PN	0	0	0
		3 PN	0	0	0
		≥4 PN	0	0	0
2 PN	62	2 PN	62	62	56*
		3 PN	0	0	0
		≥4 PN	0	0	0
3 PN	0	3 PN	0	0	0
		≥4 PN	0	0	0
≥4 PN	0	≥4 PN	0	0	0
total		64 (62 fertilized at 12 hpc)		62 (100% /fertilized)	56 (90.3% /fertilized)
# male	2				
# plug	3				

KO

hpc 12 h		15 h		36 h (1.5 d)	108 h (4.5 d)
# of eggs		# of eggs		# of eggs developing to 2-cell stage	# of eggs developing to blastocyst
UF	17	UF	16	0	0
		0 PN	1	0	0
0 PN	15	0 PN	12	0	0
		1 PN	1	0	0
		2 PN	1	1	1
		3 PN	1	1	1
		≥4 PN	0	0	0
1 PN	10	1 PN	8	6	3
		2 PN	1	1	1
		3 PN	1	1	1
		≥4 PN	0	0	0
2 PN	41	2 PN	38	38	29*
		3 PN	3	3	2
		≥4 PN	0	0	0
3 PN	20	3 PN	19	18	17
		≥4 PN	1	0	0
≥4 PN	4	≥4 PN	4	0	0
total		107 (90 fertilized at 12 hpc)		69 (76.7% /fertilized)	55 (61.1% /fertilized)
# male	4				
# plug	5				

hpc: hours post coitus. Percentages are based on the numbers of fertilized oocytes (UF; unfertilized oocytes were subtracted) at 12 h hpc. Asterisks indicate blastocyst embryos developed with retained 2PN.

Supplementary Table 2. Development of oocytes after IVF

	WT						KO					
hpi	8 h		12 h		24 h	108 h (4.5 d)	8 h		12 h		24 h	108 h (4.5 d)
	# of eggs		# of eggs		# of eggs developed to 2-cell stage	# of eggs developed to blastocyst	# of eggs		# of eggs		# of eggs developed to 2-cell stage	# of eggs developed to blastocyst
	UF	72	UF	72	0	0	UF	32	UF	28	0	0
			0 PN	0	0	0			0 PN	4	0	0
	0 PN	0	0 PN	0	0	0	0 PN	42	0 PN	39	1	0
			1 PN	0	0	0			1 PN	0	0	0
			2 PN	0	0	0			2 PN	3	3	1*
			3 PN	0	0	0			3 PN	0	0	0
			≥4 PN	0	0	0			≥4 PN	0	0	0
	1 PN	2	1 PN	2	2	1	1 PN	0	1 PN	0	0	0
			2 PN	0	0	0			2 PN	0	0	0
			3 PN	0	0	0			3 PN	0	0	0
			≥4 PN	0	0	0			≥4 PN	0	0	0
	2 PN	108	2 PN	108	97	90*	2 PN	26	2 PN	18	15	14*
			3 PN	0	0	0			3 PN	7	7	3
			≥4 PN	0	0	0			≥4 PN	1	0	0
	3 PN	3	3 PN	3	2	1	3 PN	53	3 PN	49	36	31
			≥4 PN	0	0	0			≥4 PN	4	0	0
	≥4 PN	0	≥4 PN	0	0	0	≥4 PN	54	≥4 PN	54	11	5
	total		185 (113 fertilized at 8h hpi)		101 (89.4% /fertilized)	92 (81.4% /fertilized)	total		207 (175 fertilized at 8h hpi)		73 (41.7% /fertilized)	54 (30.8% /fertilized)
	# male	3					# male	3				

hpi: hours post insemination. Oocytes were inseminated at a concentration of 1.0×10^4 sperm/ml. Percentages are based on the numbers of fertilized oocytes (UF; unfertilized oocytes were subtracted) at 8h hpi. Asterisks indicate blastocyst embryos developed from 2PN oocytes.

Supplementary Methods

Generation of Plcz1 D210R point mutant mice with the CRISPR/Cas9 system. Pronuclear injection of pX330 plasmid and reference oligonucleotides was performed to obtain mutant mice with the D210R point mutation. pX330 plasmid with an sgRNA targeting the loci (5′ï GCTGTCTGGAAATTGACTGCï 3′) and 130 mer oligonucleotide with the D210R mutation were co-injected [1]. The oligo 5′ï TATGACACTATTTTGCCTCCCTTTTGCTAGTGCTCTTGTGAAAGGCTGCCGCTGTCTGGAAAT*ACG* *T*TGCTGGGATGGATCCCAAAATGAACCCATTGTGTACCATGGTTACACATTCACCAGCAAGCTTï 3′ (mutation is italicized) was used. A founder mouse with a D210R point mutation was used to expand the colony. Genotyping was performed by DNA sequencing or restriction digestion using HpyCH4IV of PCR amplicons generated by primers a and b described above.

Generation of Plcz1 H435P point mutant mice with the CRISPR/Cas9 system. EGR-G01 ES cells were transfected with a pX330 plasmid targeting the loci (5′ï TACTCTGGTGATGAATGCCGï 3′) and a reference plasmid for homology-dependent repair (HDR) [1]. The reference plasmid was constructed in pBluescript II SK (+) vector containing the point mutation site. Homology arms with point mutations were amplified by PCR with the following primer sets {e: 5′ï GAAAGATGCTGCGCCAGAGGï 3′ and 5′ï C*T*GC*A*GT*AG*GGAAAATAAACTCATGGï 3 (mutation is italicized), 0.5 kb BamHIï PstI fragment}, or {5′ï C*T*GC*A*GCATTCATCACCAGAGTATACCï 3′ (mutation is italicized) and f: 5′ï TAGCATCAAGGAGAATCTGGï 3′, 0.5 kb PstIï XhoI fragment}. HDR and germ-line transmission were determined by DNA sequencing or by restriction digestion with PstI PCR amplicons using primers e and f above.

Testicular histology. Testes were fixed in Bouinôs fixative at 4°C overnight, then embedded into glycol methacrylate (Technobit 8100: Heraeus Kulzer, Hanau, Germany) after dehydration in a graded ethanol series. Plastic thin sections (5 µm) were treated with 1% periodic acid for 10 min, followed by treatment with Schiffôs reagent (Wako Pure Chemicals, Osaka, Japan) for 20 min. The sections were counterstained with haematoxylin prior to imaging.

Synthesis of mRNA. The modified pcDNA3.1/Myc-His vector, pcDNA3.1poly (A83), containing the open reading frame of mouse *PLCz1* was a gift from Dr. Tadashi Baba of Tsukuba University, Japan. [2]. Mutagenesis in the open reading frame was achieved using the KOD-Plus-Mutagenesis Kit (TOYOBO, Osaka, Japan) with primer sets (5′ï AATTTCCAGACAGCGGCAGCCï 3′ and 5′ï CGCTGCTGGGATGGATCCCï 3′) for D210R, (5′ï CCACCGCGGCATTCATCACCï 3′ and 5′ï GGAAAATAAACTCATGGACTCTCAATTTGGï 3′) for H435P, (5′ï CTATCCTCTAACACGCCTGACATAGï 3′ and 5′ï GCTAACAGGCAACTGGATCCCï 3′) for S536L, and (5′ï CTCTCTAACACGCCTGACATAGTAGTGï 3′ and 5′ï TGAGCTAACAGGCAACTGGATCCCï 3′) for S537L, according to the manufacturerôs instructions. Complementary DNA fragments for human *PLCZ1* were amplified by PCR using primers (5′ï GCGCGGTACCGCCGCCATGGAAATGAGATGGTTTï 3′ and 5′ï GCGCACTAGTTTATTATCTGACGTACCAAACï 3′) from a human testis cDNA library (#637209, Clontech) and inserted into a pBluescript II SK (+) vector. RNA was synthesized from linearized plasmids by T7 polymerase using the mMESSAGE mMACHINE® T7 Transcription Kit (Thermo Fisher Scientific, MA, USA), separated with phenolï chloroform, precipitated with ethanol, and dissolved in water.

Transfection of 293T cells with expression vectors. The amplified cDNA fragment of mouse *Plcz1* was introduced into a pCAG1.1 expression vector containing the CAG promoter. EGFP was introduced into the C-terminus of *Plcz1* prior to the stop codon. Transient transfection with the expression vectors was performed using the conventional calcium phosphate method. Total cell lysates were collected 48 h after transfection.

Immunoblotting. Immunoblot analysis of sperm proteins was performed as described [2] with small modifications. Cauda epididymal sperm were suspended in 10 mM Tris/HCl, pH 7.0, containing 150 mM NaCl, and 1% TritonX-100. Sperm proteins were extracted in 200 mM Tris/HCl, pH 6.8, containing 2% SDS and 10% glycerol at 100°C for 5 min, and then centrifuged at 12,000 *g* for 10 min at 4°C. Proteins in the supernatant were reduced and subjected to SDSï PAGE followed by western blotting.

Reverse transcription (RT)–PCR. Testes recovered from matured males of each genotype were dissolved in TRIzol (Ambion) and total RNAs were extracted using a guanidine isothiocyanateï phenolï chloroform method. Complementary DNA sequences were synthesized using the SuperScript III First-Strand Synthesis System (Invitrogen Life Technologies). The PCR step was performed using 100 ng of total RNA as a template for each reaction. The primers used in RTï PCR were as follows: 5′ï TTCTACAATGAGCTGCGTGTGGCCCCï 3′ and 5′ï GTGGTACGACCAGAGGCATACAGGGACï 3′ for β-actin; and 5′ï ATGGAAAGCCAACTTCATAGCTCGï 3′ and 5′ï ACATGCGTCACTCTCTGAAGTACCï 3′ for *Plcz1*.

References

1. Oji A*, et al.* CRISPR/Cas9 mediated genome editing in ES cells and its application for chimeric analysis in mice. *Sci Rep* **6**, 31666 (2016).

2. Nakanishi T*, et al.* Birth of normal offspring from mouse eggs activated by a phospholipase Czeta protein lacking three EF-hand domains. *J Reprod Dev* **54**, 244-249 (2008).
